# The Effect of Iron Deficiency Anemia Early in the Third Trimester on Small for Gestational Age and Birth Weight: A Retrospective Cohort Study on Iron Deficiency Anemia and Fetal Weight

**DOI:** 10.1155/2019/7613868

**Published:** 2019-11-22

**Authors:** Ilknur Col Madendag, Mefkure Eraslan Sahin, Yusuf Madendag, Erdem Sahin, Mustafa Bertan Demir, Banu Acmaz, Gokhan Acmaz, Iptisam Ipek Muderris

**Affiliations:** ^1^Kayseri City Hospital, Department of Obstetrics and Gynecology, Kayseri, Turkey; ^2^Department of Obstetrics and Gynecology, Erciyes University Faculty of Medicine, Kayseri, Turkey; ^3^Kayseri City Hospital, Department of Internal Medicine, Kayseri, Turkey

## Abstract

**Aim:**

The aim of the present study was to evaluate the relationship between iron deficiency anemia and small for gestational age (SGA) in early third trimester pregnancies.

**Methods:**

A total of 4800 pregnant women who met the inclusion criteria were analyzed retrospectively. We included pregnant women who had iron deficiency anemia between 26^+0^ and 30^+0^ weeks of gestation and delivered singletons between 37^+0^ and 41^+6^ weeks of gestation. Patients were divided into four groups according to anemia level: (1) hemoglobin (Hb) < 7 mg/dl (*n* = 80), (2) Hb 7–9.9 mg/dl (*n* = 320), (3) Hb 10–10.9 mg/dl (*n* = 1300), and (4) Hb > 11 mg/dl (*n* = 3100, control group). The primary outcome of this study was the presence of SGA.

**Results:**

The demographic and obstetric characteristics were similar among all the groups. Maternal age, BMI <30 kg/m^2^, nulliparity rates, and previous cesarean delivery rates were similar among groups. Ethnicity was significantly different in the severe and moderate anemia groups (<0.001). Mean fetal weight was 2900 ± 80 g in the severe anemia group, 3050 ± 100 g in the moderate anemia group, 3350 ± 310 g in the mild anemia group, and 3400 ± 310 g in the control group. Fetal weight was significantly lower in the severe and moderate anemia groups compared to the mild anemia and control groups (<0.001). The SGA rate was 18.7% in the severe anemia group, 12.1% in the moderate anemia group, 5.3% in the mild anemia group, and 4.9% in the control group. SGA was significantly higher in the severe and moderate anemia groups compared to the mild anemia and control groups (<0.001).

**Conclusion:**

The results of this study indicated that early third trimester severe and moderate iron deficiency anemia was associated with SGA. Iron deficiency anemia in pregnant women may lead to low birth weight.

## 1. Introduction

Iron deficiency anemia is the most common nutritional disorder worldwide, affecting around 40% of pregnancies [1]. Plasma volume starts to increase from the first trimester of pregnancy, which causes physiological anemia in pregnant women due to hemodilution. Hemoglobin levels are recommended by the World Health Organization (WHO) to be maintained above 11.0 g/dl in pregnancy and not to fall below 10.5 g/dl in the second trimester. According to the WHO guidelines, hemoglobin values between 10 and 10.9 g/dl are considered mild anemia, moderate anemia between 7 and 9.9 g/dl, and severe anemia below 7 g/dl [2]. In order to meet the erythrocyte requirements in the antepartum and postpartum periods, the mother needs approximately 1130 mg of total iron during this period [3]. The iron requirement is 0.8 mg/day in the first trimester and 7.5 mg/day in the last trimester [4].

Small for gestational age (SGA) is when the neonatal weight is below the 10th percentile, and it is associated with increased neonatal morbidity and mortality [5]. Some pregnancy complications such as chronic maternal diseases, premature rupture of membranes, gestational age at birth, pregestational diabetes, hypertensive disease, and multiple pregnancies may affect the degree of morbidity associated with SGA [6–11]. Fetal iron requirements increase in the third trimester, and fetal growth increases in this period. In the presence of maternal iron deficiency anemia in the early third trimester, fetal growth retardation owing to insufficient circulation and oxygenation is possible. Hence, we aimed to evaluate the relationship between iron deficiency anemia and SGA in early third trimester pregnancies.

## 2. Materials and Methods

This was a retrospective cohort study approved by the Ethics Committee of Erciyes University, Kayseri, Turkey (decision no: 2019/539), and conducted at Health Sciences University, Kayseri City Hospital, in accordance with the Declaration of Helsinki.

The study comprised 4800 pregnant women (Caucasian, native and non-Caucasian, and immigrants) who had iron deficiency anemia between 26^+0^ and 30^+0^ weeks of gestation and delivered at Kayseri City Hospital between June 2018 and July 2019. The inclusion criteria were as follows: (1) pregnant women who delivered singletons between 37 0/7 and 41 6/7 weeks of gestation, (2) the last menstrual period was used to determine the gestational week, and (3) gestational age was calculated according to ultrasonographic measurements performed in the first trimester when the last menstrual period was unknown.

The exclusion criteria were as follows: (1) pregnant women with multiple pregnancies, (2) preterm delivery before 37 weeks of gestation, (3) fetal chromosomal, or congenital anomalies, (4) tobacco, alcohol, or drug use, and (5) Hb < 4 mg/dl. A pregnancy was considered complicated if a woman had any of the following: diabetes (pregestational or gestational), pregnancy-related hypertensive disease (chronic hypertension, gestational hypertension, preeclampsia, or eclampsia), intrahepatic cholestasis of pregnancy, placenta previa, and placental abruption. All other types of anemia such as thalassemia, some chronic disease anemia, sideroblastic anemia, and megaloblastic anemia were also excluded from this study [12].

The 4800 pregnant women were divided into four groups according to anemia levels based on the WHO cutoffs [13]: (1) hemoglobin (Hb) < 7 mg/dl (*n* = 80), (2) Hb 7–9.9 mg/dl (*n* = 320), (3) Hb 10–10.9 mg/dl (*n* = 1300), and (4) Hb >11 mg/dl (*n* = 3100, control group). Iron deficiency anemia was defined when serum ferritin level was less than 15 mcg/L and infection was not present [2]. SGA was determined using the Alexander growth curve for neonatal gestational age at delivery, birth weight, and sex. SGA was defined as being below the 10th percentile according to the gestational week [14]. Anemia was classified as mild (Hb 10–10.9 g/dl), moderate (Hb 7–9.9 g/dl), or severe (Hb < 7 g/dl) based on the WHO cutoffs [13].

## 3. Statistics

Comparisons of more than two groups were made using ANOVA, followed by Tukey's *post hoc* test with Minitab 16 (MinitabInc., State College, PA, USA). For comparison of two groups, the Shapiro–Wilk test was used to determine the normality of the data and Levene's test was used to test the variance homogeneity assumption. Values are expressed as mean ± standard deviation. Parametric comparisons were made using the *t*-test, and nonparametric comparisons were made using the Mann–Whitney *U* test. The difference between the groups was considered statistically significant when *p* values were <0.05.

## 4. Results

Of the 4800 pregnant women enrolled in the study, 80 had severe anemia, 320 had moderate anemia, 1300 had mild anemia, and 3100 were healthy controls. Their demographic and obstetric characteristics were compared and are shown in Table 1. All pregnant women in the study except the control group had low serum ferritin level (<15 mcg/L). Maternal age (*p*=0.130), BMI <30 kg/m^2^ rates (*p*=0.440), nulliparity rates (*p*=0.800), and previous cesarean delivery rates (*p*=0.975) were similar among groups. Ethnicity (Caucasion and non-Caucasion) was significantly different in the severe and moderate anemia groups (*p* < 0.001).

Table 2 shows the delivery outcomes and SGA rates. Gestational age at delivery (weeks) was similar among groups (*p*=0.540). Male gender rates and vaginal delivery rates were similar among groups (*p*=0.780 and *p*=0.840, respectively). Mean fetal weight was 2900 ± 80 g in the severe anemia group, 3050 ± 100 g in the moderate anemia group, 3350 ± 310 g in the mild anemia group, and 3400 ± 310 g in the control group. Fetal weight was significantly lower in the severe and moderate anemia groups compared to the mild anemia and control groups (*p* < 0.001). Fetal weight was significantly lower in the severe anemia group compared to the other groups (<0.001). When we compared SGA rates, we found that the SGA rate was 18.7% in the severe anemia group, 12.1% in the moderate anemia group, 5.3% in the mild anemia group, and 4.9% in the control group. SGA was significantly higher in the severe and moderate anemia groups compared to the mild anemia and control groups (*p* < 0.001). In addition, SGA was significantly higher in the severe anemia group compared to the moderate anemia group (*p* < 0.001). When compared with the control group, SGA was found to be increased by 3.8-fold in the severe anemia group and 2.4-fold in the moderate anemia group.

## 5. Discussion

Anemia is an ongoing health problem during pregnancy that is prevalent worldwide. It has been estimated by the WHO that over 2 billion people, or approximately 30% of the world's population, are anemic and over 50% of pregnant women have anemia [15]. In the present study, we aimed to evaluate the relationship between iron deficiency anemia in early third trimester pregnancies and SGA; our results indicated that early third trimester moderate and severe iron deficiency anemia was associated with SGA. When compared with the control group, SGA was found to be increased by 3.8-fold in the severe anemia group and 2.4-fold in the moderate anemia group. All pregnant women in the study except the control group had low serum ferritin level (<15 mcg/L).

In the literature, there are several studies evaluating the effect of iron deficiency anemia on fetal growth and SGA but different results continue to be reported. While most of the studies focus primarily on the first and second trimesters, there are very few studies on the third trimester. It is clear that first-trimester iron deficiency anemia is associated with low fetal birth weight and SGA [16–18]. These results were supported by two recent systematic reviews [18, 19]. In the second trimester, it has been reported that iron deficiency anemia is not associated with low birth weight and SGA [19, 20]. Although the recent studies have focused on the first trimester, it is now easy to treat iron deficiency anemia, especially in the first trimester, because it is easy to meet the 0.8 mg daily iron requirement in this period compared to the second and third trimesters [1, 4]. This situation is supported by Yang et al., who reported that iron intake was associated with a reduction of SGA rates [21]. Thus, iron deficiency anemia in the first trimester is not a cause for concern regarding adverse perinatal outcomes in routine clinical practice.

In the third trimester, there have been reports that iron deficiency anemia is not related to SGA and low birth weight [19, 20], but in the present study we found that moderate and severe iron deficiency anemia in the early third trimester was associated with SGA. We may be able to explain our results with the increased fetal growth and increased iron requirements in the third trimester. For example, the fetal weight in the 50th percentile is 910 g at 26 weeks of gestation versus 3800 g at 41 weeks. There is a 4-fold increase in the fetal weight in this period, and in the presence of iron deficiency anemia fetal growth retardation is possible as a result of insufficient circulation and tissue oxygenation. In addition, it is reported that 7.5 mg/day iron is necessary in the third trimester and this requirement is approximately 10-fold higher than in the first trimester [4]. In the case of iron deficiency anemia in the early third trimester, it will not be possible to meet the iron requirement for fetal development. Although treatment with iron preparations during this period will correct anemia, it may not be possible to improve severe and moderate anemia, especially in the presence of the increased fetal requirements.

Prevention of iron deficiency anemia during pregnancy will reduce adverse maternal and perinatal outcomes. The Turkish Ministry of Health's iron support program (2007) recommends that during the nine months comprising six months starting from the second trimester and three months after birth, pregnant women should get a total of 40–60 mg of iron by considering their daily iron requirements, even if the pregnant woman has not been diagnosed with clinical anemia. Additionally, methods such as initiating the necessary treatment by monitoring ferritin levels and increasing patient compliance and motivation for daily iron and folic acid replacement intake will reduce the prevalence of iron deficiency anemia in pregnancy.

## 6. Conclusion

The results of this study indicated that early third trimester severe and moderate iron deficiency anemia is associated with SGA. When compared with the control group, SGA was found to be increased by 3.8-fold in the severe anemia group and 2.4-fold in the moderate anemia group. Iron deficiency anemia in pregnant women may lead to low birth weight. Further prospective studies with a greater number of patients are required in this regard.

## Figures and Tables

**Table 1:** Comparison of maternal characteristics.

	Severe anemia (*n* = 80)	Moderate anemia (*n* = 320)	Mild anemia (*n* = 1300)	Control (*n* = 3100)	*p* value
Maternal age (years)	25.4 ± 5.6	25.5 ± 5.5	25.8 ± 5.9	26.3 ± 5.8	0.130
BMI <30 kg/m^2^	56 (70%)	225 (70.3%)	930 (71.5%)	2171 (70%)	0.440
Nulliparity	21 (26.2%)	89 (27.8%)	370 (28.4%)	841 (26.2%)	0.800
Previous cesarean history	20 (25%)	83 (25.9%)	326 (25.0%)	766 (24.7%)	0.975
Ethnicity (Caucasian)	62 (77.4%)^a^	243 (75.9%)^a^	1146 (88.2%)^b^	2759 (89%)^b^	<0.001

Different superscripts mean statistically different. BMI, body mass index; values are expressed as mean ± SD or *n* (%).

**Table 2:** Comparison of delivery outcomes and SGA rates.

	Severe anemia (*n* = 80)	Moderate anemia (*n* = 320)	Mild anemia (*n* = 1300)	Control (*n* = 3100)	*p* value
Gestational age at delivery (weeks)	39 (38–40)	39 (38–40)	39 (38–40)	39 (38–40)	0.540
Male gender rates	43 (53.7%)	169 (52.8%)	696 (53.3%)	1677 (54%)	0.780
Vaginal delivery rates	52 (65%)	204 (63.7%)	838 (64.4%)	2021 (65.1%)	0.840
Fetal weight (g)	2900 ± 80^a^	3050 ± 100^b^	3350 ± 310^c^	3400 ± 310^c^	<0.001
SGA	15 (18.7%)^a^	39 (12.1%)^b^	70 (5.3%)^c^	152 (4.9%)^c^	<0.001

Different superscripts mean statistically different. SGA, small for gestational age; values are expressed as median (25^th^–75^th^ percentile) or mean ± SD or *n* (%).

## Data Availability

All data used for this study belong to the Kayseri City Hospital. However corresponding author also have all the data. In case of need can be obtained from the corresponding author.
